# Phylogenetic investigation of enteric bovine coronavirus in Ireland reveals partitioning between European and global strains

**DOI:** 10.1186/s13620-015-0060-3

**Published:** 2015-12-30

**Authors:** L. Gunn, P. J. Collins, M. J. O’Connell, H. O’Shea

**Affiliations:** Department of Biological Sciences, Cork Institute of Technology, Rossa Ave, Bishopstown, Cork Ireland; Bioinformatics and Molecular Evolution Group, School of Biotechnology, Dublin City University, Glasnevin, Dublin 9 Ireland

**Keywords:** Bovine coronavirus, Spike protein, Sequence analysis

## Abstract

**Background:**

Bovine coronavirus is a primary cause of neonatal calf diarrhea worldwide, and is also associated with acute diarrhea in adult cattle during the winter season. There are no reports on molecular characterization of bovine coronavirus in Ireland, and little data exists apart from serological studies.

**Findings:**

In this study, 11 neonatal (mean age 9 days) calf BCoV strains from the south of Ireland were collected over a one year period and characterized using molecular methods. The spike gene which encodes a protein involved in viral entry, infectivity and immune response shows the most variability amongst the isolates and was subsequently selected for in depth analysis. Phylogenetic analysis of the spike gene revealed that the Irish strains clustered with novel BCoV strains from Europe in a unique clade, possibly indicating lineage partitioning. Direct analysis of alignments identified amino acid changes in the spike protein unique to the Irish clade.

**Conclusion:**

Thus, monitoring of bovine coronavirus in Ireland is important as the current isolates in circulation in the south of Ireland may be diverging from the available vaccine strain, which may have implications regarding future BCoV vaccine efficacy.

## Findings

Coronaviruses (CoV, family *Coronaviridae*) are large enveloped viral particles containing a positive sense single stranded RNA genome (26–30 kb), coding for several structural proteins, including polymerase (Pol), nucleocapsid (N), membrane (M), hemagglutinin-esterase (HE), spike (S) proteins and several non-structural proteins (NSPs). Coronaviruses have been associated with respiratory and enteric infections in humans and ruminants [1].

Enteric Bovine coronavirus (BCoV) replicates in the epithelial cells of the gut, destroying villi, resulting in severe, often bloody diarrhea in calves, which can be life threatening, due to loss of electrolytes and malnutrition [2]. Disease in calves usually occurs within the first month of life, with respiratory and enteric infections being the most common conditions diagnosed. In adult cows, as a result of close confinement during transport or housing, BCoV is associated with winter dysentery and shipping fever [2]. The spike proteins of BCoV play an important role in immune response, eliciting both cellular immune responses and neutralizing antibodies [3].

BCoV has been detected in Ireland using molecular or immunological techniques [4–6], but it has not been characterized or compared to other global BCoV strains. Enteric pathogens frequently isolated from neonatal calves with enteritis in Ireland are rotavirus, cryptosporidium and much less frequently, coronavirus [7]. Currently in Ireland, a trivalent vaccine is licensed for the immunization of pregnant cows against rotavirus, coronavirus and *Escherichia coli,* confering passive immunity to calves, the coronavirus aspect of the vaccine is based on an inactivated Mebus strain. [8] In this study we aimed to: (i) characterize bovine coronavirus in the South of Ireland via analysis of the Spike gene, and (ii) compare Irish BCoV to global and vaccine isolates to identify variations in the hyper-variable region of the spike gene.

## Methods

Faecal samples were collected from the Cork Regional Veterinary Laboratory (CRVL) after they had tested positive for coronavirus using an immunochromatographic commercial kit (ICK), Corona Vet (Serosep, Ireland). Faecal samples were also screened for rotavirus, cryptosporidium and *Salmonella*. A total of 11 coronavirus positive samples were collected from neonatal calves, mean age 9 days, presenting with diarrhea between 2010 and 2011. Samples were stored at −80 °C prior to analysis.

Prior to extraction, faecal samples were homogenized in an equal volume of 0.89 % NaCl, centrifuged and filtered using a 0.20 μm pore size. The RNA was then extracted from the cell free fluid using Qiagen Viral RNA mini kit (Qiagen), following the manufacturers’ instructions. Extracted RNA was stored at −20 °C prior to analysis. Extracted RNA was tested for the presence of Coronavirus using degenerate oligonucleotide primers described previously [9], targeting a 250 bp region of the polymerase gene. A nested PCR was used to amplify the spike (S) gene [10], specifically the hypervariable region (HVR) [11].

Following analysis of this region, the most variable isolate was selected for complete characterization of the S gene using primers previously described [12, 13]. Reactions were carried out using Enhanced Avian Reverse Transcriptase kit (Sigma-Aldrich), following the manufacturers’ instructions, all reactions were carried out using a Biometra T3000 thermocycler. Amplified products were run on 1.5 % agarose gels, stained with ethidium bromide and visualized using a UV light trans-illuminator. Bands containing positive samples were cleaned using Roche High Pure PCR clean kit (Roche) and sequenced using a commercial service (MWG Eurofins, Germany).

**Fig. 1:**
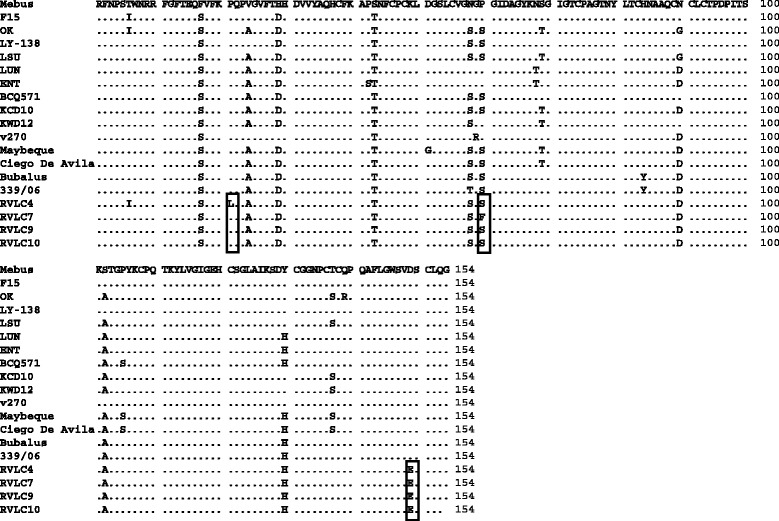
Amino acid sequence alignment profile of the hypervariable region of the spike protein. The profile was produced using Clustal W alignment and Bioedit. Amino acids in columns of the alignment with no alterations are shown as dots. A unique amino acid change occurs at position 21 in isolate RVLC4, novel alterations were detected at position 60 in RVLC7 and position 149 for all isolates

**Fig. 2:**
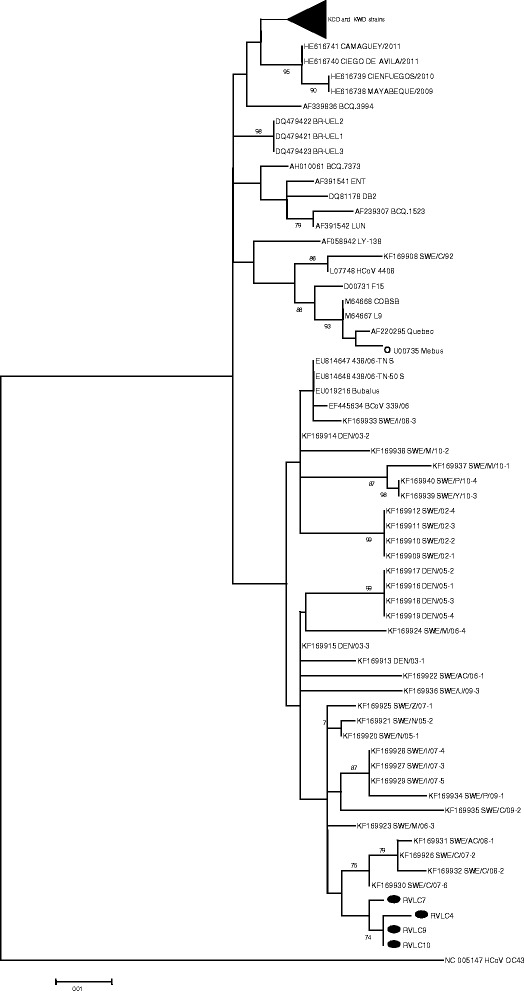
Maximum Likelihood tree based on partial length of the spike gene specifically the HVR (464 bp), nt positions 1324 to 1787. All Irish isolates from this study are represented by a filled circle (●)

**Fig. 3:**
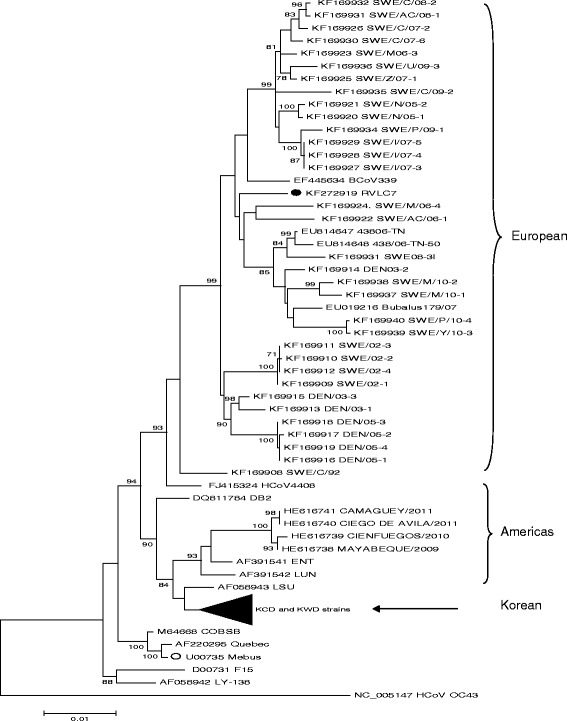
Maximum likelihood tree based on the complete spike gene (4092 bp), including the S1 and S2 subunits. Irish isolates from this study are indicated by a filled circle (●) in the tree

Resulting sequence data was then analysed and edited using Bioedit v7.0.9.0 [14] and online BLAST tool (http://blast.ncbi.nlm.nih.gov/Blast.cgi), to identify homologous strains. Sequence alignment was carried out using Clustal W in Bioedit [14], and a sequence alignment profile generated (Fig. 1). For analysis of the whole S gene, contigs were assembled using DNAstar program Seqman. The phylogenetic trees for the S gene were constructed using Maximum likelihood (ML) in MEGA5.1 [15] with a GTR model, plus gamma distribution and invariant sites with 1000 bootstrap replicates (Figs. 2 and 3). In ML trees shown, all strains are displayed with accession numbers.

Partial nucleotide sequences are available for Polymerase gene (JN204179, JN204180), Spike (KF272908, KF272910, KF272912, KF272914). The complete gene sequence of the spike protein of Irish BCoV strain RVLC7 was registered in Genbank under the accession number-KF272919.

## Results

Eleven coronavirus ICK positive specimens were collected from the CRVL and then subjected to further analysis at the Virology Unit, Department of Biological Sciences, Cork Institute of Technology (CIT). Of the 11 positive faecal samples tested using an ICK, 8/11 (72.7 %) tested positive for coronavirus using molecular techniques (Table 1). Initial analysis was carried out using the online BLAST tool, Bioedit [14], and using MEGA5.1 [15] for phylogenetic analysis. Initially, a partial sequence representing the HVR of the spike was characterized, 7/8 strains were successfully amplified, and 4 representative strains were selected and sequenced.

**Table 1:** Summary of the 11 isolates used in this study, number as per main text

			Coronavirus	
Name	Age	Year	Polymerase	Spike
RVLC1	30 days	2010	-	-
RVLC2	7 days	2010	+	+
RVLC3	5 days	2010	-	-
RVLC4	7 days	2011	+	+
RVLC5	5 days	2011	+	+
RVLC6	7 days	2011	-	-
RVLC7	7 days	2011	+	+
RVLC8	21 days	2011	-	+
RVLC9	8 days	2011	+	+
RVLC10	3 days	2011	+	+
RVLC11	9 days	2011	+	-

Sequence alignment of the spike HVR showed that Irish strains had novel substitutions compared to the Mebus strain sharing between 92.2–93.5 % amino acid identity. The hypervariable region of the spike gene (S) had 7 single nucleotide polymorphisms (SNPs) in a 464 bp sequence, resulting in 3 amino acid changes (Fig. 1); the amino acid changes occurred at positions 21, 60 and 149. Position 21 had a substitution from proline to leucine, found only in isolate RVLC4. Position 60 shows two different residue changes, the most common being proline to serine (RVLC4, 9, 10 However, only 3/4 of the Irish strains contained this proline to serine substitution, while the fourth contained a change from proline to phenylalanine (RVLC 7), which has a different polarity and lacks the ability to be phosphorylated. The position 149 shift was found in all strains and resulted in an aspartic acid residue being changed to glutamic acid here.

From alignment analysis of the HVR sequences, one isolate was selected for complete sequencing of the whole S gene, using primers previously described [12, 13]. Isolate RVLC7 was selected due to the novel polymorphism identified in position 60 of the HVR (proline to phenylalanine, corresponding to position 501 in the complete S protein). As depicted in Table 2, the complete spike protein contained a total of 14 residue changes when compared to the Mebus strain; 9 of these changes were shared with other strains found in Europe (light grey regions in Table 2), while 5 were unique to Irish isolate RVLC7 (dark grey regions in Table 2).

**Table 2:**
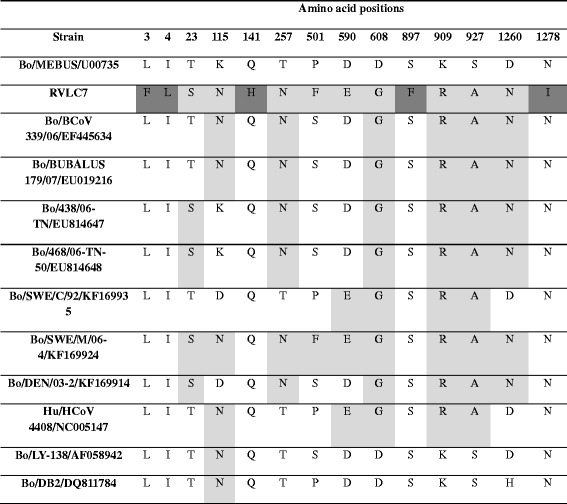
Sub-sampling of the complete alignment of the spike gene

All 14 amino acid substitutions present within the Irish isolate RVLC7 in comparison to vaccine strain Mebus are shown. Light grey regions are shared residue alterations while dark grey regions are unique to RVLC7

*Abbreviation*: *Bo* Bovine, *Hu* Human

## Discussion

In 2012, BCoV was the most commonly isolated virus from live clinical cases of pneumonia and is also an important cause of enteric disease in young Irish calves [7]. In this study, we aimed to characterize bovine coronavirus in Ireland, and compare Irish BCoV strains with field and vaccine global strains. The partial gene tree for the hypervariable region of the S1 subunit of the spike gene showed that the Irish isolates clustered together with other European isolates (Italian, Swedish and Dutch) (Fig. 2). The phylogenetic tree of the complete spike gene shows that the European isolates form a distinct clade in comparison to American, Canadian and Korean isolates these different clades may represent differences in antigenicity [16] and possibly different lineages (Fig. 3). The Irish isolates group together, clustering with European isolates, in a distinct clade. It is known that viral lineages can form natural groups based on geographical location as can be seen with human Group A Rotavirus[17]. Sequence alignment profiles of the HVR revealed a polymorphism in all Irish isolates (position 149 in Fig. 1, position 590 in the whole protein), a change from aspartic acid to glutamic acid. This change is also present in Human Coronavirus 4408, a BCoV-like isolate from a child in USA [18]. A previously identified polymorphism was identified in Irish isolate RVLC7 [11], (position 60 in Fig. 1, residue 501 in whole protein), a substitution from proline to phenylalanine was detected, this substitution has been associated immunological escape mutants through changes in protein secondary structure [12, 19]. The vaccine licensed for use in Ireland is based on the Mebus strain, which is in a different clade. This phylogenetic difference between wild type and vaccine strains has been reported previously in other jurisdictions [20]. The data presented here suggests partitioning of Irish BCoV wild type strains away from the vaccine strain, and we identified the presence of novel mutation in spike HVR in wild type strain (RVLC 7, phenylalanine 501).

## Conclusion

Monitoring of bovine coronavirus in Ireland is important as the data suggests that the current isolates in circulation maybe diverging. In addition, phylogenetic analysis demonstrated clear differences between European and global BCoV strains, therefore, continuous monitoring of BCoV is essential in order to detect new BCoV strains which may emerge and provide data which can be used to inform future BCoV vaccine design.
